# Clinical Significance of the Control CT Rotterdam Score Compared With the Admission CT Rotterdam Score in Patients With Isolated Severe Traumatic Brain Injury in the Intensive Care Unit

**DOI:** 10.7759/cureus.69792

**Published:** 2024-09-20

**Authors:** Dragan Švraka, Anita Djurdjevic Svraka, Vlado Djajic, Mile Cucak, Miso Miskic

**Affiliations:** 1 Anesthesiology and Critical Care, Faculty of Medicine, University Clinical Center of Republic of Srpska/University of Banja Luka, Banja Luka, BIH; 2 Anesthesiology, Resuscitation, and Intensive Care, University Clinical Center of Republic of Srpska/General Hospital Gradiska, Gradiska, BIH; 3 Faculty of Medicine, University of Banja Luka, Banja Luka, BIH; 4 Neurology, University Clinical Center of Republic of Srpska/University of Banja Luka, Banja Luka, BIH; 5 Radiology, University Clinical Center of Republic of Srpska/University of Banja Luka, Banja Luka, BIH; 6 Neurosurgery, University Clinical Center of Republic of Srpska/University of Banja Luka, Banja Luka, BIH

**Keywords:** neurocritical care, neuroradiology, rotterdam score, severe traumatic brain injury, trauma treatement

## Abstract

Background: The Rotterdam scale is one of the most commonly used radiological scales for evaluating and predicting outcomes in traumatic brain injury (TBI) cases. Given the evolving nature of TBI, our study is designed to compare the Rotterdam score of computed tomography (CT) findings upon admission (Rotterdam score I) with the score after 72 hours (Rotterdam score II) of treatment in the trauma intensive care unit (ICU).

Methods: A retrospective observational study was conducted on 54 patients who received intensive care treatment for isolated severe TBI over five years. We included severe TBI patients with no age restrictions who required admission to the ICU within 12 hours of the onset of trauma. An initial Rotterdam CT score was obtained via a CT head scan within four hours of the trauma, followed by a control CT head scan 72 hours after ICU admission. It was essential to have documentation on the clinical and laboratory treatment course and access to radiological CT diagnostics. Receiver operating characteristic (ROC) curves were employed in this study to evaluate the accuracy of diagnostic tests, such as the Rotterdam score. The ROC curves provided a graphical representation of the tests' diagnostic performance, which helped assess their effectiveness.

Results: There was a significant difference (p < 0.001) in the diagnostic scores of CT scans upon admission (Rotterdam score I) and control CT scans after 72 hours (Rotterdam score II) in the total sample. The Rotterdam score I was notably higher, 3.6 (±0.8), in patients requiring neurosurgical intervention compared to those who did not, 2.8 (±0.9), with significance (p = 0.003). The Rotterdam score I demonstrated a substantial predictive value for unfavorable outcomes (p = 0.048), as did the Rotterdam score II after the 72-hour mark (p = 0.006).

Conclusion: The control Rotterdam score 72 hours after admission predicts mortality in isolated TBI patients more significantly than the Rotterdam score determined at the patient's admission to the intensive care unit.

## Introduction

Both developing and developed countries face the same issue of traumatic injuries. According to the World Health Organization (WHO), nearly 90% of trauma-related deaths occur in low- or middle-income countries, where 85% of the world's population lives [1]. One of the most prevalent causes of trauma-related fatalities is traumatic brain injury (TBI), which often results in early deaths and is frequently the cause of hospital admission [2]. Around 10% of TBI patients require intensive care unit (ICU) admission, and not all experience isolated head trauma [3]. Brain injuries are classified into three levels of severity: mild, moderate, and severe. This classification is determined using scoring systems based on clinical examinations or radiological diagnostics. It is important to note that the estimated degree of injury can fluctuate within the first 24-72 hours following trauma, particularly when considering the mechanisms of injury. Several methods exist for performing brain imaging during this dynamic phase at various intervals. Assessing the severity of a TBI can be subjective and context-dependent, as it is often influenced by the experience and reference point of the assessor. While the Glasgow Coma Scale is commonly used to distinguish between moderate and severe cases, it may provide only a snapshot at the early stages of assessment. A TBI initially classified as moderate may still require the same level of sophisticated surveillance, monitoring, and treatment as a severe form or may even progress to a severe, challenging-to-treat condition [4]. The medical procedures used for treating TBI in the ICU have remained largely unchanged over the last two decades, and the scientific evidence supporting most of these interventions is weak [5]. Given the dynamic nature of TBI, this study aims to compare the Rotterdam score of computed tomography (CT) findings at admission (Rotterdam score I) with the score after 72 hours (Rotterdam score II) of treatment in the ICU. We seek to determine whether evaluating the Rotterdam score from control CT findings after 72 hours of treatment in the ICU offers better predictive value for survival outcomes.

## Materials and methods

A retrospective observational study was carried out on patients who received treatment for isolated severe TBI over five years at the largest trauma Intensive Care Unit of the Clinic for Anesthesia and Intensive Care at the University Clinical Centre in the Republic of Srpska, Bosnia and Herzegovina (ICU UCCRS). Patient data were collected by reviewing printed and electronic medical records, utilizing the Clinical Information System and the Radiological Information System. All 54 patients selected to participate in the study underwent a clinical examination by an experienced anesthesiologist and neurosurgeon and were classified as having severe TBI, with a Glasgow Coma Score (GCS) of 3-8, before being admitted to the ICU. All study TBI patients received treatment according to the established protocol. Upon admission to the ICU, CT scans were conducted and repeated after 72 hours of treatment. An experienced neuroradiologist evaluated the CT diagnostic images taken during admission and follow-up, determining the Rotterdam scores.

Established ICU protocol for severe TBI patients

Upon admission, patients were placed on controlled mechanical ventilation with a thiopental infusion to induce a barbiturate coma, maintained at a minimum dose of 4 g every 12 hours until a control CT scan of the head was performed. In addition to barbiturates, anti-edema therapy and opioids were administered as required. Monitoring involved invasive arterial line measurements without invasive intracranial pressure monitoring. Neurological examinations were conducted every six hours.

Inclusion criteria

Isolated severe TBI (GCS 3-8) with no age restrictions requiring admission to the ICU within 12 hours of the onset of trauma. An initial Rotterdam CT score was obtained via a CT head scan within four hours of the trauma, followed by a control CT head scan 72 hours after ICU admission. It was essential to have documentation on the clinical and laboratory treatment course and access to radiological CT diagnostics.

Exclusion criteria

Traumatic injury to other organs or systems, admission to the ICU beyond 12 hours from the initial trauma, the requirement for extensive blood transfusions, a history of prior head injury or neurosurgery, incomplete medical records, or the inability to obtain a clear understanding of radiological CT scans.

Statistical data processing

**Figure 1:**
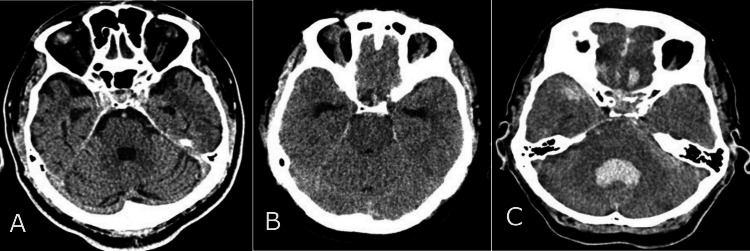
Basal cisterns (BC) CT findings CT normal findings of BC (A); compressed BC (B); absent BC (C).

**Figure 2:**
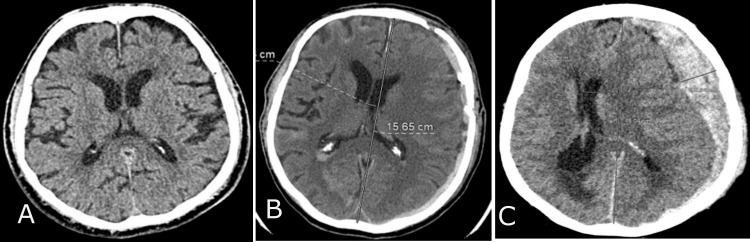
Midline shift CT findings Midline shift normal findings (A); midline shift ≤5 mm (B); midline shift >5 mm (C).

**Figure 3:**
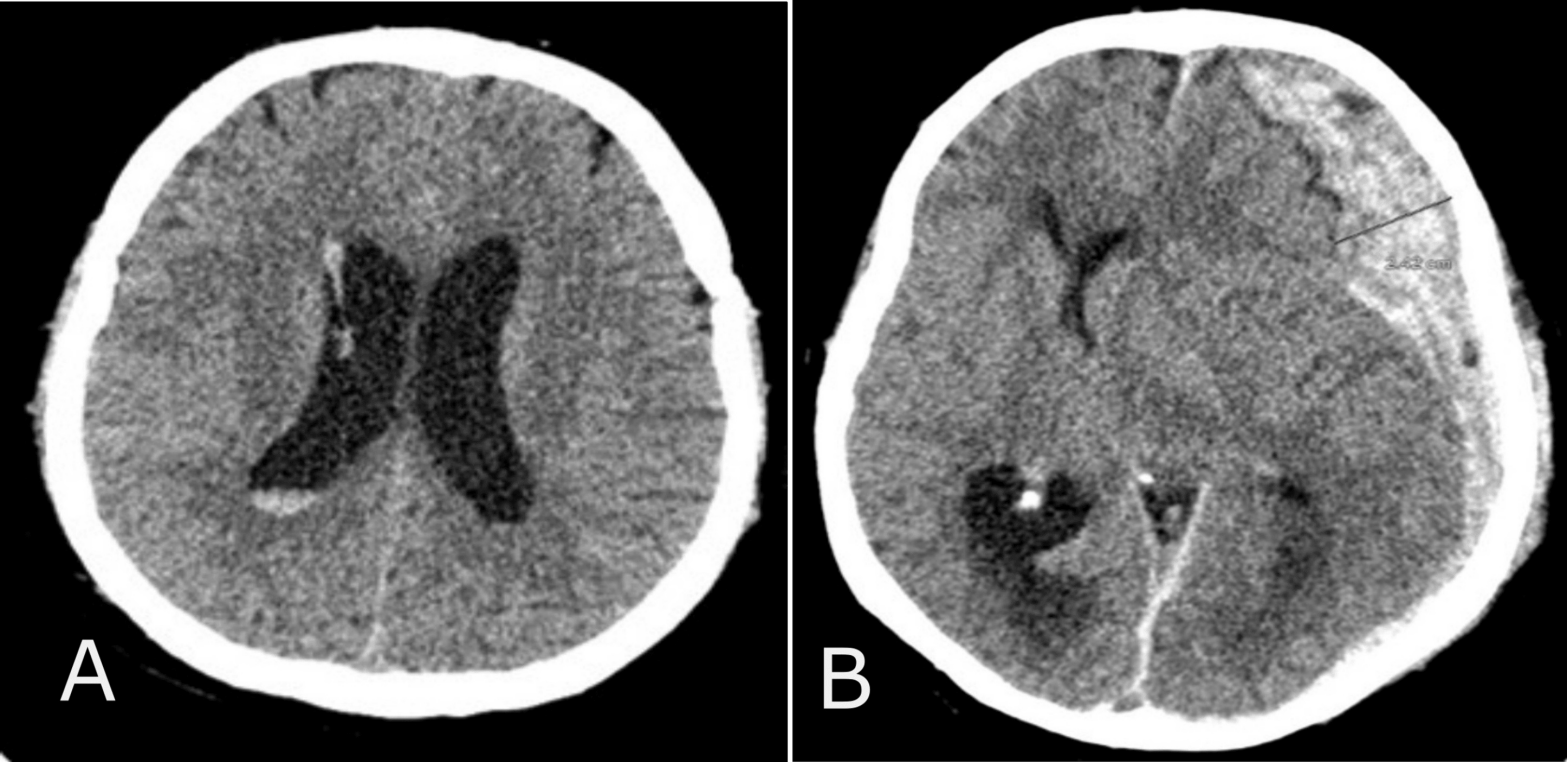
Epidural mass lesion CT findings Epidural space normal findings (A); epidural mass lesion acute epidural hematoma (B).

**Figure 4:**
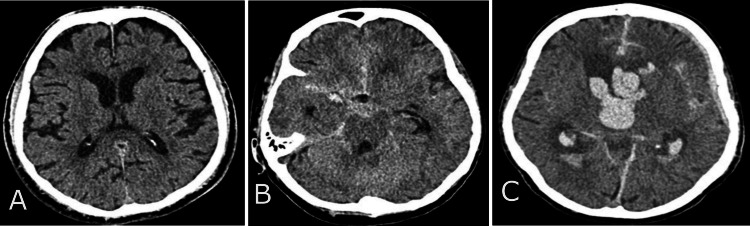
Intraventricular blood or traumatic subarachnoidal haemorrhage (SAH) CT findings Intraventricular and subarachnoidal space normal findings (A); subarachnoidal hemorrhage (SAH) (B); intraventricular hemorrhage (IVH) and SAH (C).

The study monitored patient data, including age, gender, etiological factors, and mortality rates, using valid observations per group (n) and percentage (%). The etiological factors that led to trauma are divided into three categories: patients injured in traffic vehicles, patients injured in traffic as pedestrians, and those injured from accidental falls and crash injuries. The median was used to present the descriptive data, with the interquartile range (IQR) as variance, and age was reported in years. The Rotterdam CT score of TBI is a classification aimed at improving prognostic evaluation of patients admitted with moderate or severe traumatic brain injuries. The continuous variables, Rotterdam scores, include four independently scored elements. They include (1) the degree of the basal cistern (BC) compression (normal findings of BC; compressed BC; absent BC) (Figure 1) and (2) the degree of midline shift (midline shift normal findings (A); midline shift ≤ 5mm (B); midline shift > 5mm (C)) (Figure 2). It does not include contusions but restricts mass lesions to (3) epidural hematomas (epidural space normal findings (A); epidural mass lesion acute epidural hematoma (B)) (Figures 3) and adds (4) intraventricular and subarachnoid hemorrhage (intraventricular and subarachnoidal space normal findings (A); subarachnoidal hemorrhage (SAH) (B); intraventricular hemorrhage (IVH) and SAH (C)) (Figure 4). Each of these is given a score, and these scores are tallied, with one added to the total. In other words, an utterly normal-appearing scan has a Rotterdam score of 1, and the worst possible score is 6. Classification of Rotterdam score goes basal cisterns (0: Normal, 1: compressed, 2: absent); midline shift (0: no shift or ≤F 5 mm; 1: shift > 5 mm); epidural mass lesion (0: present, 1: absent); and intraventricular blood or traumatic SAH (0: absent; 1: present).

The statistical analysis of the entire group was conducted by determining the mean, standard deviation (±SD), and T-test (with significance < 0.05, 95% CI) for both the Rotterdam score at admission and the control Rotterdam score to assess the significance of any differences between the two scores.

Receiver operating characteristic (ROC) curves were employed in this study to evaluate the accuracy of two diagnostic tests, Rotterdam Score I and Rotterdam Score II, in identifying severe TBI outcomes among patients admitted to the ICU. The ROC curves provided a graphical representation of the tests' diagnostic performance, helping to assess their effectiveness. By comparing the diagnostic value of each test, the study determined which was more effective in predicting severe TBI outcomes.

Statistical data processing was performed using IBM SPSS Statistics for Windows, Version 20 (Released 2011; IBM Corp., Armonk, New York).

## Results

In patients with severe TBI, males were more prevalent, accounting for 75% (n = 41) of the population, while females accounted for 25% (n = 14). The most common cause of injury was traffic trauma, with victims in vehicles comprising 52% (n = 29) of cases. Pedestrians injured in traffic accounted for 24% (n = 13), while other causes, such as accidental falls and crash injuries, also accounted for 24% (n = 13). In the ICU, patients with severe isolated TBI had a mortality rate of 18% (n = 10) (Table 1).

The patients observed with isolated severe TBI had a median age of 39, ranging from 35 to 47 years. Men had a median age of 40 years, with a range of 35 to 47 years, while women tended to be younger, with a median age of 29 years and a range of 21 to 48 years. In cases of traffic trauma, the youngest population had a median age of 33 years, ranging from 22 to 48 years. The median age for pedestrian victims was 51 years, with a range of 7 to 64 years, while other victims had a median age of 44 years, ranging from 41 to 63 years. Deceased patients were mostly in their sixth decade, with a median age of 54 years, ranging from 24 to 63 years (Table 1).

**Table 1:** General characteristics of the isolate severe TBI study population This table presents the general characteristics of the study population with isolated severe traumatic brain injury (TBI), including the number of valid observations per group (n), percentage (%), age (in years), central tendency (median) with dispersion (interquartile range, IQR), and a 95% confidence interval (CI 95%). The variables include gender, age, mechanism of injury, and mortality.

Descriptive characteristics	Severe TBI patient, n (%)	Age median (IQR)
All study patients	55 (100%)	39 (24-56)
Male	41 (75%)	40 (35-47)
Female	14 (25%)	29 (21-48)
Vehicle accident	29 (52%)	33 (22-48)
Pedestrian	13 (24%)	51 (7-64)
Other mechanisms of trauma	13 (24%)	44 (41-63)
Mortality	10 (18%)	54 (24-63)

In Table 2, it was found that there was a significant difference (p < 0.001) in the diagnostic scores of CT scans upon admission (Rotterdam score I) and control CT scans after 72 hours (Rotterdam score II) in the total sample. Out of the 55 patients diagnosed with isolated severe TBI, 16 (29%) underwent neurosurgical procedures within the first 24 hours. The Rotterdam score I was notably higher, at 3.6 (±0.8), in patients requiring neurosurgical intervention compared to those who did not, at 2.8 (±0.9), with significance (p = 0.003). Interestingly, the Rotterdam score II showed a declining trend in all patients from admission, with no significant difference observed between those who received neurosurgical treatment and those who did not (p = 0.36). These scores were represented as means of values (±SD).

**Table 2:** Rotterdam scores were measured on admission (Rotterdam score I) and 72 hours later (Rotterdam score II) in the total sample as between surgically and non-surgically treated patients to determine neurological damage and recovery Rotterdam scores are presented as means of values, with a variance of standard deviation mean (±SD). *t-test (95% CI; significance <0.05).

Surgery treatment data	Rotterdam score I (mean ±SD)	Rotterdam score II (mean ± SD)	p*
Total (n = 55, 100%)	4.1 (±0.9)	3.4 (±1)	<0.001
Neuro-surgically treated (n = 16, 19%)	3.6 (±0.8)	2.6 (±0.8)	0.003
Non-surgically treated (n = 39, 71%)	2.8 (±0.9)	2.3 (±1)	0.36

The CT diagnostic Rotterdam score test has shown potential in forecasting the prognosis of patients with severe isolated TBI. The test is conducted twice, once upon admission and again after 72 hours. Specifically, the Rotterdam score I demonstrated substantial predictive value for unfavorable outcomes (p = 0.048), as did the Rotterdam score II after the 72-hour mark (p = 0.006). These results are extensively elaborated, along with the logistic regression findings (area under the ROC curve), in Table 3.

**Table 3:** The predictive value of CT findings in two Rotterdam scores for diagnostic purposes Rotterdam score l (CT findings at admission in ICU); Rotterdam score II (CT findings after 72 hours in ICU); AUC (area under the ROC curve). *The asymptotic significance of the ROC curve is less than 0.05, with a confidence level of 95%.

CT findings – Rotterdam scores	AUC	p*
Rotterdam score l	0.7	0.048
Rotterdam score ll	0.8	0.006

**Figure 5:**
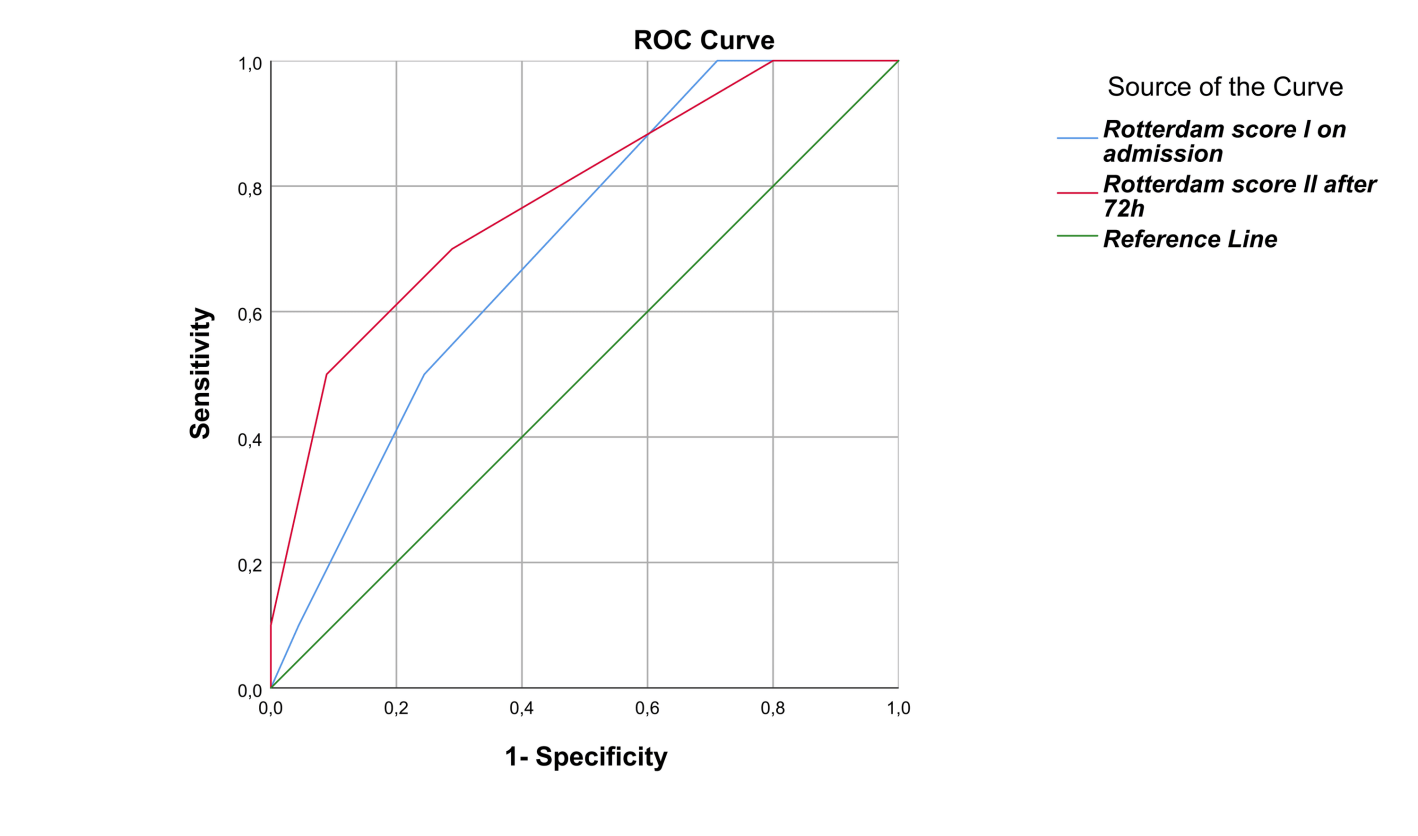
Receiver operating characteristic (ROC) curves: a plot of sensitivity versus (1 - specificity) The asymptotic significance of the ROC curve is less than 0.05, with a confidence level of 95%.

Based on the analysis, it has been determined that the Rotterdam score II curve, denoted by the red curve, exhibited the highest AUC value of 0.781 compared to other curves. This indicates that it possesses a superior actual positive rate (sensitivity) and serves as a more precise diagnostic test for outcomes than the Rotterdam score I curve, represented by the blue curve, which had an AUC value of 0.701 (Figure 5; Table 3).

## Discussion

According to research, the effects of TBI can vary based on location and are often influenced by factors such as living conditions and cultural norms [6]. In medium-developed countries, traffic accidents are nearly three times as likely to cause TBI compared to highly developed countries [1]. Recent studies conducted in the USA, Europe, and Japan over the last decade have shown a decline in TBI resulting from traffic accidents, while incidents caused by falls in individuals over 65 have increased [6-8]. It is important to note that interpreting these findings can be complicated by varying legal regulations related to traffic safety and road quality throughout Europe.

It can be challenging to compare the age and gender distribution of TBI patients due to incomplete data in many studies. However, we have endeavored to avoid limitations beyond the prevalence of severe TBI and have focused solely on the five-year outcomes from a single hospital. According to a comprehensive European study [9], males make up the majority of TBI patients across the continent, though the exact proportion varies. In this study of traumatic brain injuries, the lowest mean age was reported in the Republic of San Marino at 26.7 years, and the highest was reported in Austria at 44.5 years.

CT is the primary imaging tool used for the initial triage and follow-up of acute TBI. It is recommended that a first CT scan be obtained quickly after TBI occurs. However, the current decision-making process for determining whether or not to perform an initial CT scan is inefficient. Up to 95% of scanned patients show no intracranial injury and are unnecessarily subjected to radiation risks [10]. As per a review study on neurosurgery, the time taken to obtain a CT scan does not seem to impact mortality rates significantly [11]. Similarly to Goswami et al.'s study [12], it is worth mentioning that the absence of any CT abnormalities does not necessarily imply a lack of structural damage. The Rotterdam score is a valuable tool in cases involving the management of mild traumatic brain injuries or legal procedures, as well as in situations where CT scans do not provide a complete picture of the severity of the clinical condition. This scoring system, developed in 2006, assesses CT findings of the endocranium and is gaining recognition. It is intended for the prognostic evaluation of patients admitted with moderate and severe head trauma. As in other studies, we observed a statistically significant association between ICU mortality and the Rotterdam score from the initial CT findings [13]. A large retrospective observational study that examined the prediction of in-hospital mortality using the Marshall and Rotterdam scores found that these CT scoring systems have independent prognostic value in TBI, even in alcoholic patients [14]. The CT Rotterdam score has also been recommended for predicting outcomes in pediatric TBI patients [15].

Determining when to conduct a control CT of the head in patients with TBI is a multifaceted issue, as the injury is ever-changing and leaves many uncertainties. The significance of this matter is amplified by the fact that the external forces that cause TBI can result in various types of injuries within the same brain. Ideally, the uncertainty in predicting long-term outcomes due to pathophysiological factors should be reflected in the injury classification, to avoid inappropriate and potentially nihilistic or overly optimistic clinical decision-making [16]. For all these reasons, we analyzed the Rotterdam score of control CT findings after 72 hours of ICU treatment. Our findings indicate that, following 72 hours of ICU treatment, there was a notable reduction in the average value of the control Rotterdam score. However, the ROC curve analysis revealed that the CT Rotterdam score exhibited greater sensitivity in predicting the outcome of TBI in the ICU after 72 hours of trauma than the Rotterdam score taken upon admission to the ICU.

Many researchers frequently investigate the outcomes and prognosis of patients who have suffered from TBI. Researchers use various parameters that are not typically available to clinicians to gain insights into possibilities for improving the treatment of patients with this complex injury [17]. Our research explores the potential for predicting ICU treatment outcomes based on daily diagnostic methods. Evaluating TBI patients' survival and treatment results outside of the ICU can be quite challenging due to the varying parameters that can impact their recovery [18].

Limitations and weaknesses of the study

Certain limitations to this study could impact the reliability of our research findings. The retrospective analysis relies solely on the data obtained from medical histories sourced from a single medical center. Additionally, the patient sample size is relatively small, limited to those with isolated TBI, and evaluated as severe TBI by both an anesthesiologist and a neurosurgeon. Assessing the severity of TBI in an acute care setting can be complicated by various factors, including language barriers, inexperienced evaluators, and the effects of drugs on a patient's level of consciousness. Additionally, it is essential to consider the retrospective nature of data collection, as it can lead to potential sampling errors.

## Conclusions

TBI is a complex and dynamic life-threatening injury. A CT scan is a cost-effective and noninvasive diagnostic modality. Repeated CT scans in TBI patients can assist in planning care and predicting prognosis outcomes. Our study revealed that the Rotterdam score calculated after 72 hours of ICU treatment is more sensitive in predicting outcomes than the Rotterdam score calculated at the time of the patient's admission to the intensive care unit.
